# Structure and Mechanical Performance of Poly(vinyl Alcohol) Nanocomposite by Incorporating Graphitic Carbon Nitride Nanosheets

**DOI:** 10.3390/polym11040610

**Published:** 2019-04-03

**Authors:** Shaojian He, Jiaqi Wang, Mengxia Yu, Yang Xue, Jianbin Hu, Jun Lin

**Affiliations:** 1 Beijing Key Laboratory of Energy Safety and Clean Utilization, North China Electric Power University, Beijing 102206, China; heshaojian@ncepu.edu.cn (S.H.); jqw9542@163.com (J.W.); ymx63162331@163.com (M.Y.); naoh2160@hotmail.com (J.H.); 2 State Key Laboratory of Multi-phase Complex Systems, Institute of Process Engineering, Chinese Academy of Sciences, Beijing 100190, China

**Keywords:** poly(vinyl alcohol), graphitic carbon nitride, nanosheets, crystallinity, mechanical performance

## Abstract

Owing to the high aspect ratio, the two-dimensional (2D) inorganic nanofillers have attracted extensive interest in the field of polymer reinforcement. In this work, graphitic carbon nitride (g-C_3_N_4_) nanosheets were obtained via thermal condensation of melamine and were then ultrasonically exfoliated in water, which was confirmed by atomic force microscopy (AFM) and TEM. Poly(vinyl alcohol) (PVA)/g-C_3_N_4_ nanocomposites were achieved by solution casting using water as the solvent. The structure and mechanical performance of PVA/g-C_3_N_4_ nanocomposites were studied. It was found that the g-C_3_N_4_ nanosheets were well dispersed in the PVA matrix. The introduction of g-C_3_N_4_ nanosheets increased the glass transition temperature and crystallinity of the nanocomposites, leading to the improved mechanical performance. Compared with the pure PVA, the PVA/g-C_3_N_4_ nanocomposite with 0.50 wt% g-C_3_N_4_ nanosheets showed ~70.7% enhancement in tensile strength, up from 51.2 MPa to 87.4 MPa.

## 1. Introduction

Polymer nanocomposites are among the most important materials in the academic and industrial fields, and are produced by dispersing into the polymeric matrix with nanofillers that have one or more dimensions at nano-scale. Filler dispersion in a polymer matrix is crucial to obtain high-performance nanocomposites [1,2,3]. Enhancements in the performance of the final nanocomposites depends largely on the morphological aspects of these fillers, such as their sizes and shapes. Among the nanofillers, two-dimensional (2D) nanofillers, including layered silicate, layered double hydroxide (LDH), boron nitride (BN), graphene and graphene oxide (GO), have attracted extensive interest due to the high aspect ratio [4,5,6,7,8,9,10,11,12,13]. Compared to the bulk polymers, the polymer nanocomposites filled with 2D nanofillers usually exhibit dramatically different or superior overall performance.

Over the past few years, increasing attention has been paid to graphitic carbon nitride (g-C_3_N_4_) nanosheets, a promising 2D nanomaterial with a graphene-like structure, which can be synthesized easily, rapidly and inexpensively. The g-C_3_N_4_ nanosheets have been utilized in many research areas [14,15,16,17,18,19], which are, however, mostly limited in the field of photocatalysis and heterogeneous catalysis. Recently, Zhu et al. [20] reported that the wear loss of the composite was reduced by introducing g-C_3_N_4_ as a filler into poly(vinylidene difluoride) (PVDF) matrix. Gang et al. [21] prepared sulfonated poly(ether ether ketone)/g-C_3_N_4_ nanocomposite membrane with a reduced methanol permeation. Although g-C_3_N_4_ has been used as fillers incorporated into some polymers to improve their performance, the application of g-C_3_N_4_ in polymer reinforcement remained rarely explored.

It is expected that the mechanical performance of the polymer can be positively improved by the introduction of g-C_3_N_4_ nanosheets, because the structure of g-C_3_N_4_ is similar to that of graphene. Moreover, g-C_3_N_4_ nanosheets are easily dispersed in water to form stable aqueous suspension due to the weak van der Waals force between the nanosheets [19]. Therefore, in our work, 2D ultrathin g-C_3_N_4_ nanosheets were obtained via thermal condensation and were then ultrasonically exfoliated in water, and poly(vinyl alcohol) (PVA)/g-C_3_N_4_ nanocomposites were achieved by environmental-friendly solution blending. The structures of g-C_3_N_4_ nanosheets and PVA/g-C_3_N_4_ nanocomposite were analyzed, and the mechanical performance of the nanocomposites were studied to evaluate the effect of using g-C_3_N_4_ as the filler for performance improvement of polymer composites.

## 2. Experimental Section

### 2.1. Materials

Melamine was purchased from Guangfu Chemical Research Institute, Tianjin, China. PVA (1788) was supplied by Aladdin, Shanghai, China.

### 2.2. Sample Preparation

**Figure 1:**
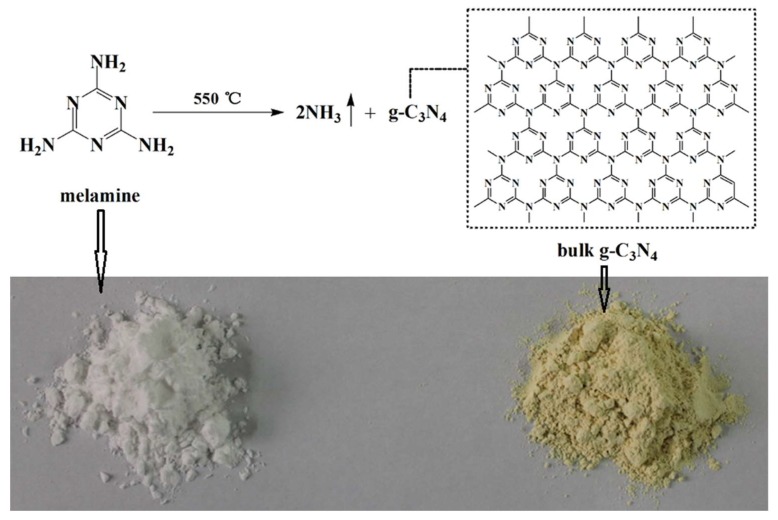
Thermal condensation process from melamine to bulk g-C_3_N_4_.

Melamine, covered by a tin foil paper in a muffle furnace, was heated to 550 °C at the heating rate of 10 °C/min and maintained at 550 °C for 2 h. After being cooled in air, the yellow product bulk g-C_3_N_4_ was obtained (as illustrated in Figure 1), which was milled into the powder and then dispersed in water with a stirring rate of 13,000 rpm for 30 min. After ultrasonic exfoliation for 48 h, the mixture was left to sit still for 36 h to remove unexfoliated g-C_3_N_4_ particles, yielding the stable aqueous suspension of g-C_3_N_4_ nanosheets (~1 mg/mL in concentration).

**Figure 2:**
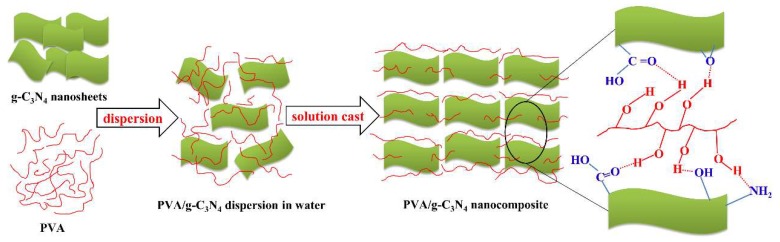
Schematic illustration of nanocomposite preparation and the interaction between g-C_3_N_4_ nanosheets and poly(vinyl alcohol (PVA).

PVA was dissolved in deionized water at 80 °C for 3 h and then mixed with the aqueous suspension of g-C_3_N_4_ nanosheets. The mixture was decanted into a glass dish and dried in an oven at 80 °C for 36 h, and then dried under vacuum at 60 °C for 12 h to thoroughly remove the water. Finally, the prepared film (~60 μm in thickness) was carefully peeled off from the dish to obtain PVA/g-C_3_N_4_ nanocomposite (as illustrated in Figure 2).

### 2.3. Measurements

Atomic force microscope (AFM) images were obtained from a SPM-9500 AFM (Shimadzu, Kyoto, Japan) (the dilute dispersions of the samples were drop-cast onto the freshly cleaved silicon surface). Transmission electron microscopy (TEM) images were recorded by a JEM 2010 EX microscope (JEOL, Tokyo, Japan) at an accelerating voltage of 200 kV. Scanning electron microscope (SEM) images were acquired from a JSM-7001F microscope (JEOL) with an acceleration voltage of 20 kV. Differential scanning calorimetry (DSC) experiments were conducted under a nitrogen atmosphere using a STARe system DSC (Mettler-Toledo Co., Schweiz, Switzerland) at a heating rate of 5 °C·min^−1^. The mechanical behavior was characterized according to ISO 527-3-1995 (specimen type 2) using an AI-7000S1 electrical tensile tester (Goodtechwill Testing Machines, Co. Ltd., Qingdao, China) at a speed of 2 mm·min^−1^.

## 3. Results and Discussion

### 3.1. Characterization of g-C_3_N_4_ Nanosheets

**Figure 3:**
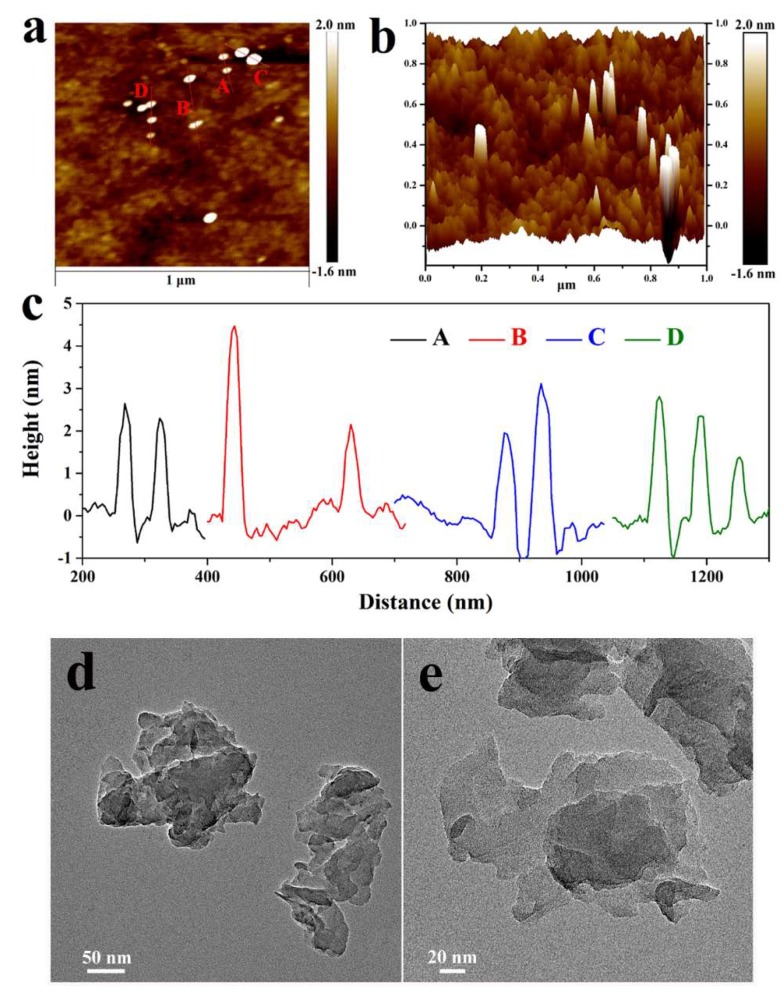
Atomic force microscope (AFM) (**a**) top view, (**b**) height topography image, (**c**) height trace curves of g-C_3_N_4_ nanosheets placed on a silicon substrate, and (**d**,**e**) TEM images of g-C_3_N_4_ nanosheets.

The transformation from melamine to g-C_3_N_4_ was confirmed by XRD, FTIR and XPS, as shown in the supporting information (Figures S1 and S2). The morphologies of the as-prepared g-C_3_N_4_ nanosheets were observed by AFM and TEM. In the AFM images shown in Figure 3, the thickness of the nanosheets is measured to be 2.0~4.5 nm, indicating that the bulk g-C_3_N_4_ was successfully exfoliated into ultrathin nanosheets. Based on the AFM images, the size of the g-C_3_N_4_ nanosheets is evaluated to be 50–80 nm, which is also supported by TEM observation. As shown in Figure 3d,e, the as-prepared g-C_3_N_4_ nanosheets consist of stacks of the nanosheets.

### 3.2. SEM Observation of PVA/g-C_3_N_4_ Nanocomposites

**Figure 4:**
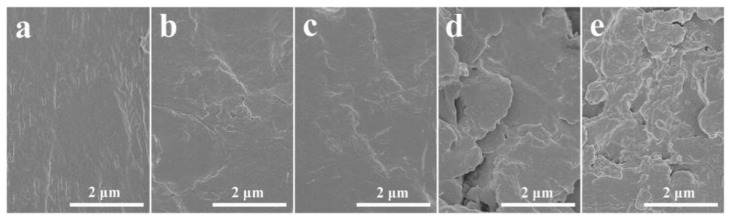
SEM images of tensile fractured surface for (**a**) pure PVA and PVA/g-C_3_N_4_ nanocomposites with g-C_3_N_4_ content of (**b**) 0.25 wt %, (**c**) 0.50 wt %, (**d**) 0.75 wt % and (**e**) 1.00 wt %.

As shown in Figure 4a–c, the similar morphologies are observed for the PVA and PVA/g-C_3_N_4_ nanocomposites with g-C_3_N_4_ content of 0.25 wt% and 0.50 wt%, indicating that the g-C_3_N_4_ nanosheets are well embedded in the matrix of these two nanocomposites. As illustrated by XPS (Figure S2), there exist –OH, –NH_2_ and –COOH on the surface of g-C_3_N_4_ nanosheets, which could form hydrogen bonding with the –OH groups on PVA macromolecules (as illustrated in Figure 2). As a result, the interfacial interaction would be quite strong in the PVA/g-C_3_N_4_ nanocomposites, leading to the good filler dispersion in the PVA matrix. However, as seen in Figure 4d,e, some g-C_3_N_4_ aggregates are exposed on the fractured surface of the nanocomposites, indicating the deteriorated filler dispersion in the matrix when more than 0.50 wt% g-C_3_N_4_ nanosheets are added. Moreover, voids are observed in the nanocomposites with g-C_3_N_4_ content of 0.75 wt% and 1.00 wt%, demonstrating the severe stress concentration and poor stress transfer in these nanocomposites caused by the filler aggregates.

### 3.3. XRD of PVA/g-C_3_N_4_ Nanocomposites

**Figure 5:**
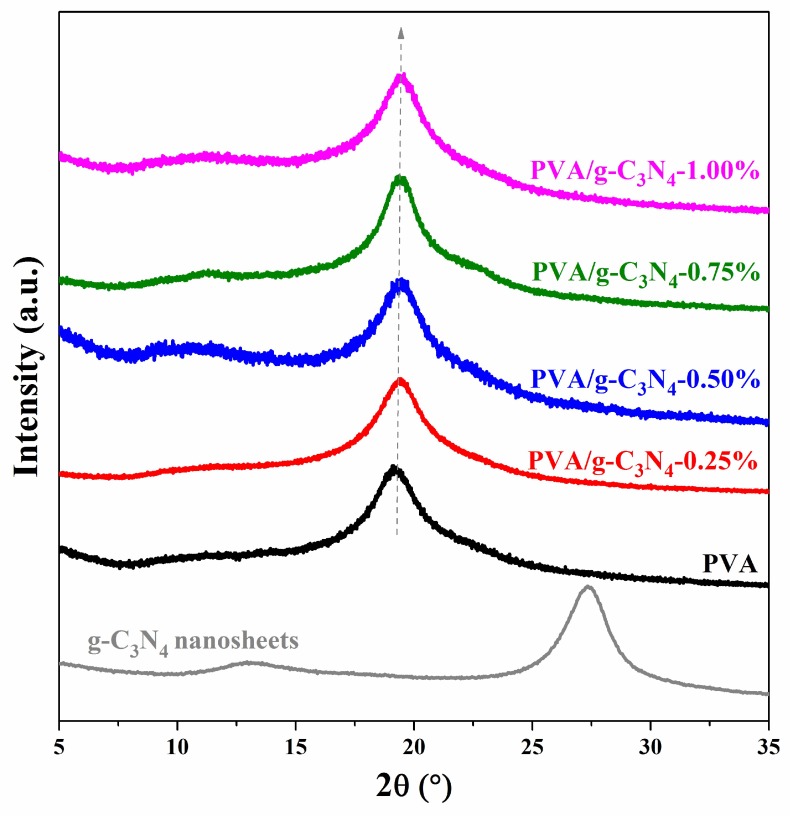
XRD curves of PVA/g-C_3_N_4_ nanocomposites with various g-C_3_N_4_ contents.

XRD curves of g-C_3_N_4_ nanosheets, pure PVA and PVA/g-C_3_N_4_ nanocomposites with various g-C_3_N_4_ contents are shown in Figure 5. As a typical semi-crystalline polymer, the diffraction peak at 19.5° for the pure PVA should be due to the crystalline phase of the polymer [7]. The XRD patterns of PVA/g-C_3_N_4_ nanocomposites with various g-C_3_N_4_ are similar to that of pure PVA, suggesting that the incorporation of g-C_3_N_4_ nanosheets into the PVA matrix will not dramatically change the crystal structure of PVA. In addition, the diffraction peaks at 27.7° and 12.8° associated with g-C_3_N_4_ nanosheets disappear, which should be due to the relatively low content of filler in the nanocomposites.

### 3.4. DSC Analysis of PVA/g-C_3_N_4_ Nanocomposites

**Figure 6:**
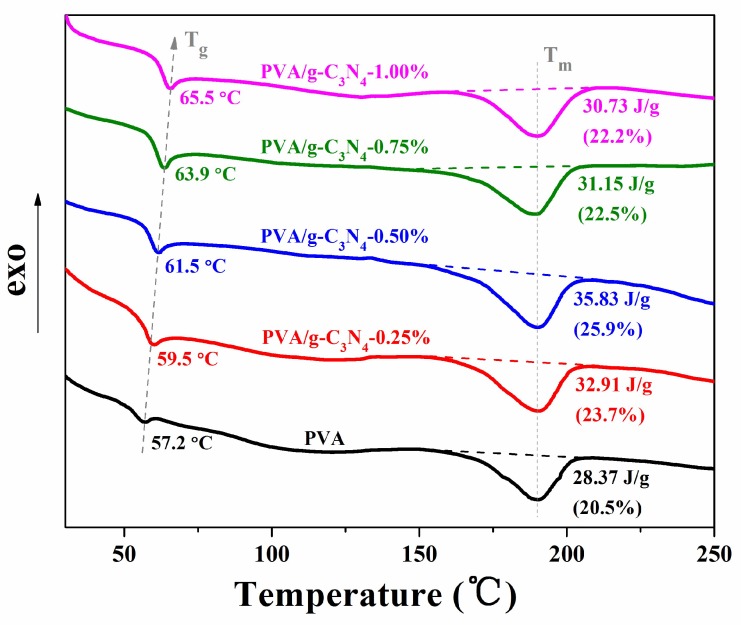
DSC curves of pure PVA and PVA/g-C_3_N_4_ nanocomposites.

The glass transition temperature (T_g_), melting temperature (T_m_) and melting enthalpy (ΔH_m_) of the pure PVA and PVA/g-C_3_N_4_ nanocomposites were obtained from the DSC curves, as shown in Figure 6. It was found that the T_g_s of the PVA/g-C_3_N_4_ nanocomposites were all higher than that of pure PVA and increased with the increasing g-C_3_N_4_ content. By adding only 1.00 wt% g-C_3_N_4_, the T_g_ significantly increased from 57.2 °C for pure PVA to 65.5 °C for the nanocomposite. Such an increase should be ascribed to the strong mobility restriction of PVA chain segments by the g-C_3_N_4_ nanosheets. Moreover, as shown in Figure 6, there exhibits little difference for the T_m_ between the pure PVA and PVA/g-C_3_N_4_ nanocomposites. By taking 138.6 J/g as the melting enthalpy for the perfect crystalline PVA [22], the calculated crystallinities of the nanocomposites are illustrated in Figure 6. With the increase of g-C_3_N_4_ content, the crystallinity of PVA/g-C_3_N_4_ nanocomposites first increases until reaching a maximum of 25.9% at 0.50 wt% g-C_3_N_4_ content and then dropped to 22.2% at 1.00 wt% g-C_3_N_4_ content, still higher than that of pure PVA (20.5%). Such results may be rationalized as follows: the increased crystallinity for the composites with a relatively low content of nanoparticles is often observed, as widely reported in the literature [23,24,25], because the small number of nanoparticles, serving as nucleating agents, could promote polymer crystallization. However, when more g-C_3_N_4_ is incorporated, these nanosheets might gather to form aggregates and weaken their promotion effect on the PVA crystallization, leading to a slight decline in crystallinity. Therefore, the crystallinity of PVA/g-C_3_N_4_ nanocomposites first rises and then declines with the increasing g-C_3_N_4_ content.

### 3.5. Mechanical Performance of PVA/g-C_3_N_4_ Nanocomposites

**Figure 7:**
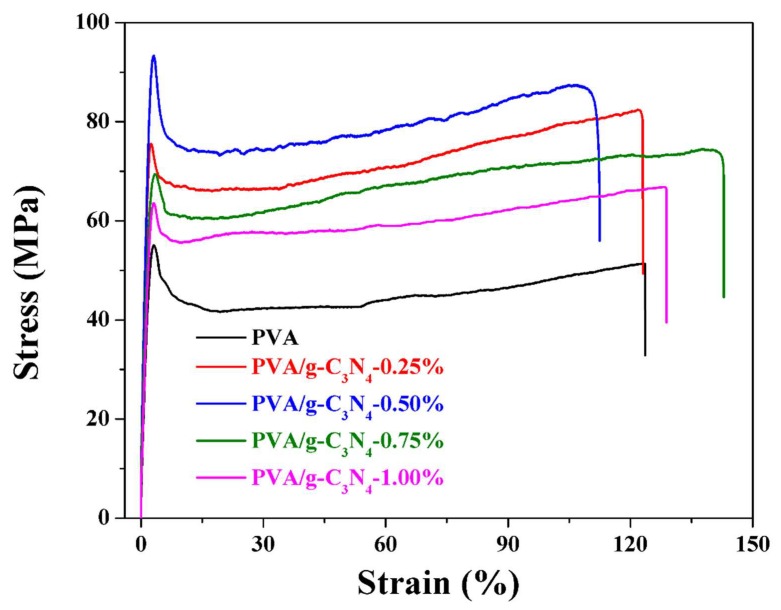
Stress–strain curves of pure PVA and PVA/g-C_3_N_4_ nanocomposites.

The mechanical performance for pure PVA and PVA/g-C_3_N_4_ nanocomposites is presented in Table 1, and the stress–strain curves for these nanocomposites are shown in Figure 7. Compared to those of the pure PVA, the elastic modulus, yield strength and tensile strength of the PVA/g-C_3_N_4_ nanocomposite with g-C_3_N_4_ content of 0.5 wt% increase by ~66.7%, ~69.5% and ~70.7%, respectively, while the elongation at break declines by ~8.9%. With further increasing g-C_3_N_4_ content to 1.00 wt%, the elastic modulus, yield strength and tensile strength slightly decrease, but still higher than those of pure PVA. Usually, higher crystallinity corresponds to the higher elastic modulus and strength. Therefore, the change of mechanical performance of PVA/g-C_3_N_4_ nanocomposites is similar to that of the crystallinity as a function of the g-C_3_N_4_ content. The PVA/g-C_3_N_4_ nanocomposite containing 0.50 wt% g-C_3_N_4_ has the highest crystallinity, leading to the strongest elastic modulus and strength. When the applied strain beyond the yield strain, the irreversible forced high-elastic deformation takes place, which originates from the forced motion of the polymeric chain segments under stress. For PVA/g-C_3_N_4_ nanocomposites, such motion may be restricted by the presence of g-C_3_N_4_ nanosheets. In addition, the good dispersion of g-C_3_N_4_ nanosheets in the nanocomposites with a relatively low content from 0.25 wt% to 0.50 wt% also results in the good stress transfer, facilitating the excellent reinforcement. Moreover, as shown in Table 2, ~70.7% improvement of the tensile strength in our work is comparable with or even higher than those of the PVA nanocomposites filled with various 2D nanofillers in the previous reports. Therefore, the g-C_3_N_4_ nanosheets exhibit an exciting potential as the filler for the reinforcement of polymeric materials.

## 4. Conclusions

In this work, an attempt has been made to evaluate the effect of using g-C_3_N_4_ nanosheets on the mechanical performance of polymer composites. After thermal condensation of melamine, the as-prepared bulk g-C_3_N_4_ were ultrasonically exfoliated in water to form a stable aqueous suspension of g-C_3_N_4_ nanosheets. The successful exfoliation of g-C_3_N_4_ nanosheets was observed by AFM and TEM. The mixture of aqueous PVA solution and g-C_3_N_4_ nanosheets suspension was cast to prepare the PVA/g-C_3_N_4_ nanocomposites. As demonstrated by SEM, the g-C_3_N_4_ nanosheets were well dispersed in the PVA matrix. Moreover, by introducing g-C_3_N_4_ nanosheets in the PVA matrix, the nanocomposites exhibited the higher glass transition temperature and crystallinity as compared to the pure PVA, resulting in the improved mechanical performance. Therefore, the present study demonstrates that the g-C_3_N_4_ nanosheets could be applied as a promising filler to effectively reinforce polymer to achieve high-performance.

## Figures and Tables

**Table 1:** Mechanical performance of pure PVA and PVA/g-C_3_N_4_ nanocomposites.

Content of g-C_3_N_4_ (wt %)	0	0.25	0.50	0.75	1.00
Elastic modulus (GPa)	2.28 ± 0.12	3.66 ± 0.17	3.80 ± 0.14	2.62 ± 0.09	2.48 ± 0.08
Yield strength (MPa)	55.1 ± 1.7	75.6 ± 2.1	93.4 ± 3.8	69.4 ± 1.9	63.6 ± 2.2
Tensile strength (MPa)	51.2 ± 2.8	82.3 ± 3.2	87.4 ± 2.6	74.3 ± 1.9	66.8 ± 2.3
Elongation at break (%)	124 ± 8	123 ± 7	113 ± 5	143 ± 11	129 ± 7

**Table 2:** Comparison of the improvement in tensile strength for the PVA nanocomposites filled with 2D nanofillers.

Filler	Content (wt%)	Tensile strength (MPa)		Improvement (%)	Reference
		Pure PVA	Nanocomposite		
graphene ^a^	3.0	17.0	42.0	~147	[26]
	0.5		27.0	~58.8	
graphene ^b^	1.8	33.5	113	~237	[7]
	0.7		67.6	~101	
	0.3		65.0	~94.0	
GO	2.0	22.5	45.7	~103	[6]
	0.5		32.1	~42.7	
BN	0.8	77.0	91.0	~18.2	[27]
BN	2.0	46.0	99.2	~115	[28]
	0.5		81.5	~77.1	
LDH	1.0	58.9	114	~93.0	[8]
	0.5		88.1	~49.6	
LDH ^b^	2.0	28.3	47.0	~66.0	[29]
MoS_2_	5.0	84.0	105	~24.0	[30]
montmorillonite	1.0	~62.0	~68.5	~10.5	[31]
g-C_3_N_4_	0.5	51.2	87.4	~70.7	Our work

^a^ The mass fraction was converted from the volume fraction according to the related density mentioned in the reference; ^b^ the filler was modified by the organic component.

## Supplementary Materials

The following are available online at https://www.mdpi.com/2073-4360/11/4/610/s1, Figure S1: (a) XRD and (b) FTIR curves of melamine, bulk g-C3N4 and g-C3N4 nanosheets; Figure S2: XPS (a) survey scan, (b) N1s, (c) C1s and (d) O1s of g-C3N4 nanosheets.
