# Supplementary material for: Structure and Mechanical Performance of Poly(vinyl Alcohol) Nanocomposite by Incorporating Graphitic Carbon Nitride Nanosheets
Source: Polymers (Basel). 2019 Apr 3;11(4):610. doi: 10.3390/polym11040610 (PMC6523783; doi:10.3390/polym11040610)
Supplement: Supplementary file 1 [file polymers-11-00610-s001.pdf]

Supporting information

Structure and mechanical performance of poly(vinyl alcohol) nanocomposite by incorporating graphitic carbon nitride nanosheets

Shaojian He [a], Jiaqi Wang [a], Mengxia Yu [a], Yang Xue [b,*], Jianbin Hu [a], Jun Lin [a,*]

[a] Beijing Key Laboratory of Energy Safety and Clean Utilization, North China Electric Power University, Beijing, 102206, China

[b] State Key Laboratory of Multi-phase Complex Systems, Institute of Process Engineering, Chinese Academy of Sciences, Beijing, 100190, China

* Corresponding author.

E-mail: xueyang198510@sina.com (Yang Xue); jun.lin@ncepu.edu.cn (Jun Lin)

Measurements

X-ray diffraction (XRD) analysis was performed using a D/Max-III C diffractometer (Rigaku, Japan) with CuKa radiation. The chemical structure of the samples was characterized by a TENSOR27 Fourier transformed infrared spectroscopy (FTIR) (Bruker Optik GMBH, Germany). The chemical composition of the samples was characterized by an ESCALAB 250 X-ray photoelectron spectroscopy (XPS) (Thermo Fisher Scientific, USA) with a 150W monochromatic Al Kα radiation.

Characterization of g-C_3N_4 nanosheets

The structure of the g-C_3N_4 nanosheets were characterized via XRD and FTIR, respectively. As shown in Figure S1a, The strong (002) peak of bulk g-C_3N_4 at 27.7°

(0.326 nm) is contributed from the interlayer stacking of the conjugated aromatic systems, whereas the (100) peak at 12.8° (0.675 nm) is attributed to the in-plane structural packing motif [1]. In contrast, the g-C_3N_4 nanosheets exhibit a significantly decreased (002) peak and a less pronounced (100) peak, suggesting that the bulk g-C_3N_4 has been exfoliated successfully. However, the unchanged peak positions indicate that the layered structure still exists in the nanosheets with the same interlayer distance. The FTIR spectra are quite similar for the bulk g-C_3N_4 and the g-C_3N_4 nanosheets, as shown in Figure S1b. The peaks at 807 cm^{-1}, 1700~1200 cm^{-1}, and 3500~3000 cm^{-1} are related to the breathing mode of the s-triazine ring, the typical skeletal stretching vibrations of s-triazine derivatives such as bridging C–NH–C or trigonal (N–$(C)_3$) units, and the stretching vibrations of the residual N–H or O–H, respectively [2, 3].

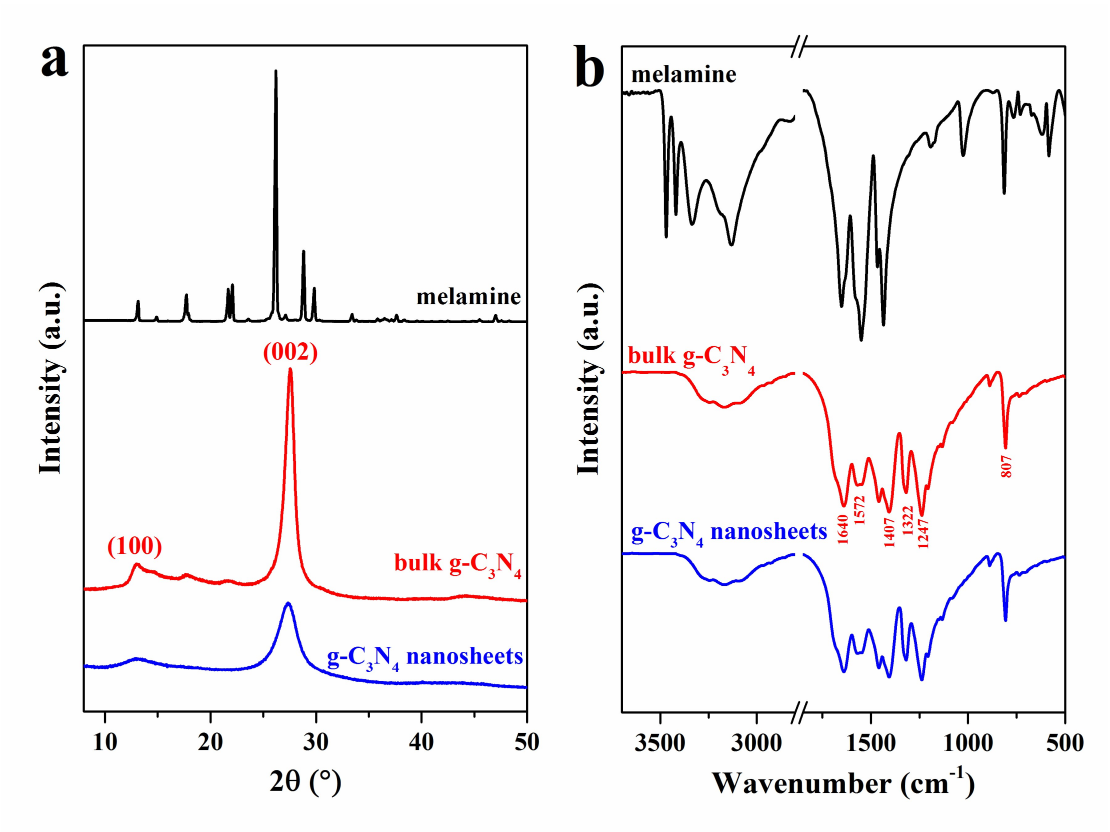

Figure S1 (a) XRD and (b) FTIR curves of melamine, bulk g-C_3N_4 and g-C_3N_4

nanosheets

To investigate the chemical state of the elements in g-C_3N_4 nanosheets further, XPS measurement was conducted. As shown in Figure S2a, nitrogen, carbon and oxygen are observed in the g-C_3N_4 nanosheets, and the calculated ratio of N/C in g-C_3N_4 nanosheets is 1.74, close to the theoretical value of 1.56. As shown in Figure S2b, three peaks at 398.5 eV, 399.8 eV and 401.1 eV, separated from the main peak of the N1s spectrum, are related to sp^2 hybridized nitrogen atoms in triazine rings (C–N=C), bridging nitrogen atoms (N–$(C)_3$) and amino groups (C–NH_2), respectively [4, 5]. The other peak at 404.4 eV is due to the charging effect [4, 6]. Meanwhile, the C1s spectrum in Figure S2c shows three peaks located at 288.1 eV, 286.1 eV and 284.4 eV. The peak at 288.1 eV should be assigned to sp^2 hybridized carbon in C–N=C [6, 7], while the low peak at 289.1 eV is attributed to the C–O bonds [5]. The peak at 284.4 eV is due to carbon contamination [8], while the low peak at 286.1 eV originates from the C–NH_2 species [5]. Moreover, as shown in Figure S2c, three peaks at 530.7 eV, 531.8 eV and 533.1 eV are found in the O1s spectrum, contributed from O=C–OH, C=O and C–OH groups, respectively [5]. The XPS results indicate that there exist a certain amount of defects, including –OH, –NH_2 and –COOH, on the surface of g-C_3N_4 nanosheets, which would help improve the compatibility between the PVA matrix and g-C_3N_4 nanosheets through the formation of hydrogen bonding.

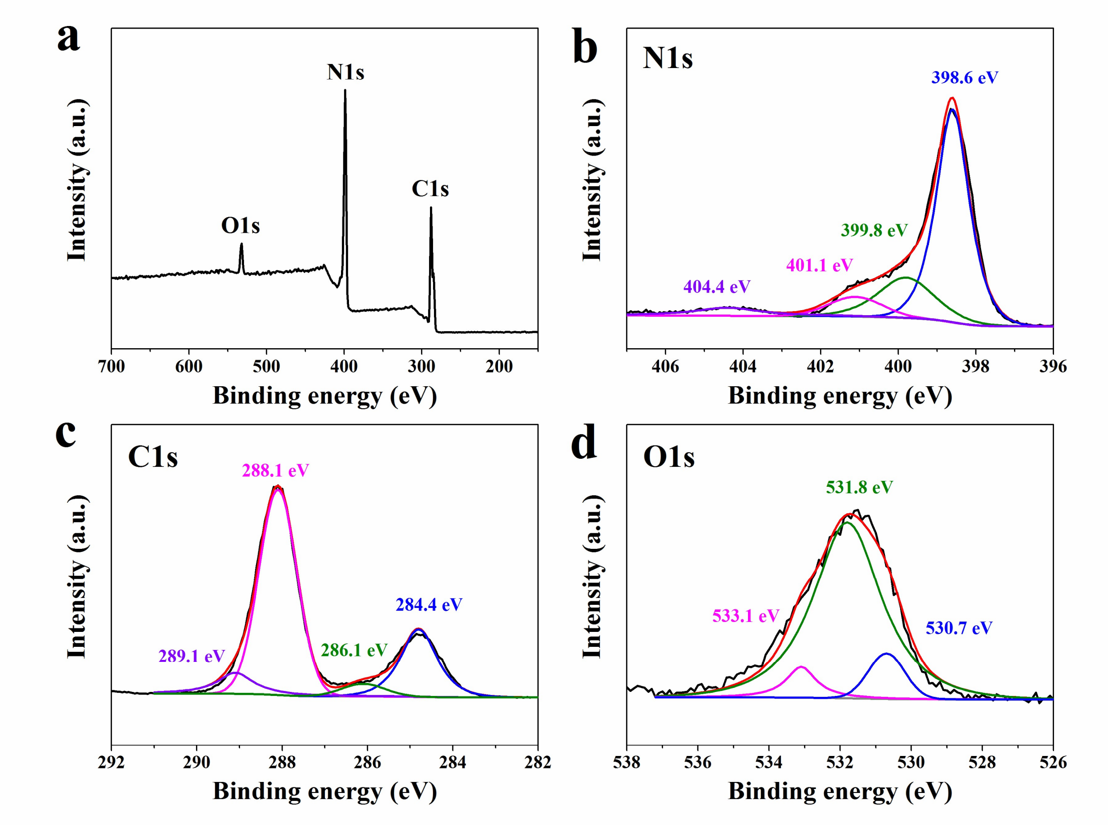

Figure S2 XPS (a) survey scan, (b) N1s, (c) C1s and (d) O1s of g-C_3N_4 nanosheets

References

[1] Z. Wang, W. Guan, Y. Sun, F. Dong, Y. Zhou, W.-K. Ho, Water-assisted production of honeycomb-like g-CN with ultralong carrier lifetime and outstanding photocatalytic activity, Nanoscale, 7 (2015) 2471-2479.

[2] S. Yang, Y. Gong, J. Zhang, L. Zhan, L. Ma, Z. Fang, R. Vajtai, X. Wang, P.M. Ajayan, Exfoliated Graphitic Carbon Nitride Nanosheets as Efficient Catalysts for Hydrogen Evolution Under Visible Light, Adv. Mater., 25 (2013) 2452-2456.

[3] X. Ding, J. Zhu, Z. Yue, X. Qian, W. Bi, X. Yang, J. Yang, Separation and concentration of natural products by fast forced adsorption using well-dispersed velvet-like graphitic carbon nitride with response surface methodology optimisation, Talanta, 154 (2016) 119-126.

[4] Qing, Zhao, Chuangang, Lingxiao, Zhipan, Zhang, Chen, Liangti, Facile production of ultrathin graphitic carbon nitride nanoplatelets for efficient visible-light water splitting, Nano Res., 8 (2015) 1718-1728.

[5] H.J. Li, B.W. Sun, L. Sui, D.J. Qian, M. Chen, Preparation of water-dispersible porous g-C_3N_4 with improved photocatalytic activity by chemical oxidation, Phys. Chem. Chem. Phys., 17 (2015) 3309-3315.

[6] J. Tong, L. Zhang, F. Li, K. Wang, L. Han, S. Cao, Rapid and high-yield production of g-C_3N_4 nanosheets via chemical exfoliation for photocatalytic H_2 evolution, RSC Adv., 5 (2015) 88149-88153.

[7] B. Song, T. Wang, H. Sun, H. Liu, X. Mai, X. Wang, L. Wang, N. Wang, Y. Huang, Z. Guo, Graphitic carbon nitride (g-C_3N_4) interfacially strengthened carbon fiber epoxy composites, Compos. Sci. Technol., 167 (2018) 515-521.

[8] K. Li, L. Yan, Z. Zeng, S. Luo, X. Luo, X. Liu, H. Guo, Y. Guo, Fabrication of $H_3PW_{12}O_{40}$-doped carbon nitride nanotubes by one-step hydrothermal treatment strategy and their efficient visible-light photocatalytic activity toward representative aqueous persistent organic pollutants degradation, Appl. Catal. B-Environ., 156-157 (2014) 141-152.
